# Implementation of ultra-hypofractionated radiotherapy schedules for breast cancer during the COVID-19 pandemic in the Netherlands

**DOI:** 10.1016/j.ctro.2024.100807

**Published:** 2024-06-14

**Authors:** Anouk H. Eijkelboom, Marcel R. Stam, Desirée H.J.G. van den Bongard, Margriet G.A. Sattler, Enja J. Bantema-Joppe, Sabine Siesling, Marissa C. van Maaren

**Affiliations:** a Department of Health Technology and Services Research, Technical Medical Centre, University of Twente, Drienerlolaan 5, 7522 NB, Enschede, the Netherlands; b Department of Research and Development, Netherlands Comprehensive Cancer Organisation (IKNL), Boven Clarenburg 2, 3511 CV, Utrecht, the Netherlands; c Radiotherapiegroep, Wagnerlaan 47, 6815 AD Arnhem, the Netherlands; d Department of Radiation Oncology, Amsterdam UMC, Meibergdreef 9, 1105 AZ, Amsterdam, the Netherlands; e Department of Radiotherapy, Erasmus MC Cancer Institute, University Medical Centre Rotterdam, Dr. Molewaterplein 40, 3015 GD Rotterdam, the Netherlands; f Department of Radiation Oncology, Radiotherapy Institute Friesland, Borniastraat 36, 8934 AD Leeuwarden, the Netherlands

**Keywords:** Radiotherapy, Breast cancer, Clinical guideline

## Abstract

**Background and purpose:**

The COVID-19 pandemic resulted in an accelerated recommendation to use five-fraction radiotherapy schedules, according to the FAST- and FAST-Forward trial. In this study, trends in the use of different radiotherapy schedules in the Netherlands were studied, as well as the likelihood of receiving five fractions.

**Materials and methods:**

Data from the NABON Breast Cancer Audit-Radiotherapy and Netherlands Cancer Registry was used. Women receiving radiotherapy for their primary invasive breast cancer or DCIS between 01–01-2020 and 31–12-2021 were included. Logistic regression was used to investigate the association between patient-, tumour-, treatment-, and radiotherapy institution-related characteristics and the likelihood of receiving five fractions in tumours meeting the FAST and FAST-Forward criteria.

**Results:**

Detailed information about radiotherapy treatment was available for 9,392 tumours. Shortly after the start of the COVID-19 pandemic, i.e. April 2020, 19% of the tumours being treated with radiotherapy received five fractions of 5.2 or 5.7 Gray (Gy). While only 3% of the tumours received five fractions in March 2020. The usage of five fractions increased to 26% in December 2021. Partial breast irradiation, compared to whole breast irradiation, was significantly associated with the administration of five fractions, as well as radiotherapy delivered in an academic radiotherapy institution compared to an independent institution.

**Conclusion:**

The start of the COVID-19 pandemic was associated with the early use of ultra-hypofractionated radiotherapy schedules. After publication of the trials, and mainly after the recommendation by the national radiotherapy society, the implementation further increased. These schedules were not yet used in all patients meeting the eligibility criteria for the FAST- or FAST-Forward trial.

## Highlights

• The COVID pandemic led to the early use of ultra-hypofractionated radiotherapy.
• Use of 5 fractions increased after recommendation by the Dutch radiotherapy society in December 2020.
• 5 fractions were mostly given in partial breast irradiation.
• Academic institutions started using 5 fractions before independent institutions.

## 1 Introduction

Radiotherapy has been shown to reduce the risk of recurrences and breast cancer deaths in patients treated with breast-conserving surgery, and in patients who receive a mastectomy with a high-risk of recurrence [1], [2]. Until 2010 the conventional radiotherapy dose fractionation schedule consisted of 50 Gray (Gy) in 25 fractions, over five weeks. In 2010 and 2013 three randomized controlled trials showed that 42.5 Gy in 16 fractions [3], 41.6 Gy or 39 Gy in 13 fractions, and 40 Gy in 15 fractions were at least as safe and effective as 50 Gy in 25 fractions [4], [5], [6]. Besides, switching from a 25-fraction to a 15-fraction schedule reduced treatment time, and proved to be more cost-effective and patient-friendly [7], [8], [9]. After publication of these results, randomized controlled trials on ultra-hypofractionation (five-fraction schedules) were started [10], [11].

Between April 2020 and August 2020 the results of three trials on ultra-hypofractionation were published [10], [11], [12]. The five-year results of the FAST-Forward trial showed that 26 or 27 Gy in five once-daily fractions over one week was non-inferior to 40 Gy in 15 fractions in terms of ipsilateral breast tumour recurrence risk [11]. An increased risk of any moderate or marked normal tissue effects was observed for patients receiving 27 Gy. According to the FAST trial the 10-year risk of late normal tissue effects did not differ between women receiving 28.5 Gy in five once-weekly fractions over five weeks and women receiving 50 Gy in 25 fractions. A higher risk was found for those receiving 30 Gy [10]. Additionally, the 10-year results of the Florence trial showed no difference in ipsilateral breast cancer recurrence risk between patients receiving partial breast irradiation (PBI), with a schedule of 30 Gy in five once-daily fractions, and those receiving whole-breast irradiation, with a schedule of 50 Gy in 25 fractions followed by a boost [12].

The COVID-19 pandemic, starting in February/March 2020 in Europe, resulted in an accelerated recommendation to use the ultra-hypofractionated radiotherapy schedules. In March 2020, a group of researchers, including European opinion leaders and researchers from the FAST and FAST-Forward trials, formulated a set of international guidelines on radiotherapy for breast cancer patients during the COVID-19 pandemic. In one of these guidelines the use of the ultra-hypofractionated radiotherapy schedules was suggested for patients with node-negative tumours that do not require a boost [13]. These recommendations were based on preliminary results of the FAST and FAST-Forward trials [14], [15], [16], combined with the knowledge of the researchers of the FAST and FAST-Forward trial regarding the outcomes of their trials. The five-fraction schedules were meant to reduce the risk of COVID-19 transmission and the pressure on the radiotherapy capacity, by limiting the number of visits to the hospital*.*

Little is known on the implementation of the five-fraction schedules, and whether certain patients had an increased chance of receiving these. Therefore, the aim of the current study was to investigate trends in the use of different radiotherapy schedules in 2020/2021 in the Netherlands. Moreover, the association between patient-, tumour-, treatment- and radiotherapy institution-related characteristics and the chance of receiving a certain radiotherapy schedule was investigated.

## 2 Materials and methods

### 2.1 Patients

Data was obtained from the NABON Breast Cancer Audit-Radiotherapy (NBCA-R) and Netherlands Cancer Registry (NCR). The primary goal of the NBCA-R is to monitor the quality of breast cancer radiotherapy [17]. The NBCA-R includes information on radiotherapy treatment (extracted from the Digital Imaging and Communication in Medicine (DICOM) radiotherapy data), including dosimetry and fractionation schedules, and was established in 2020. The first year 13 of the 19 Dutch radiotherapy institutes entered data into the NBCA-R. This increased to 18 in 2023. The NCR is a nationwide population-based registry, including records of all newly diagnosed malignancies notified through the Dutch Nationwide Pathology Databank (Palga). It includes data on patient, tumour (e.g. pathology, staging) and treatment characteristics.

Patients included in the NBCA-R (either invasive tumours or DCIS) with a radiotherapy planning CT between 01–12-2019 and 31–12-2021 were linked to the NCR. All patients treated with radiotherapy between 01–01-2020 and 31–12-2021 were eligible for inclusion. The start date of radiotherapy according to the NBCA-R was taken unless: the patient was not included in the NBCA-R, the start date was missing, or the start date was inaccurate (i.e. start date of the second tumour was taken for the first tumour or vice versa). In these cases the start date according to the NCR was used.

Two cohorts were generated. Cohort A was used to investigate the comparability between tumours included and not included in the NBCA-R, and to investigate the completeness of the NBCA-R. This cohort consisted of patients entered both in the NBCA-R and the NCR, and patients only included in the NCR. Cohort B was used to investigate the trends in the use of different radiotherapy schedules, and the likelihood of receiving a certain radiotherapy schedule. This cohort only consisted of patients entered both in the NBCA-R and NCR.

Tumours were excluded if: they were only registered in the NBCA-R but not in the NCR, they were diagnosed in males, they were not treated with surgery, it concerned a distant metastasis, the other breast was synchronously (within ≤ 91 days) irradiated, there was a history of radiotherapy on the ipsilateral breast, it concerned a recurrence. If a patient was diagnosed with a synchronous tumour in the same breast, the tumour with the highest pathological tumour stage was included.

### 2.2 Definitions

Four schedules of (local or locoregional) radiotherapy were defined: 15 fractions without boost, 20 fractions with boost (including concurrent and sequential boost), 5 fractions without boost, and other. Three periods were defined: pre-COVID (January 1st till March 15th, 2020), first COVID-year (March 16th till December 31st, 2020), and second COVID-year (January 1st till December 31st, 2021). The separation between the ‘first’ and ‘second’ COVID-year was chosen because in December 2020 five fractions of 5.2 Gy was recommended as standard treatment in the Netherlands for patients aged ≥ 50 years, receiving breast only radiotherapy without boost, during a meeting of the National Platform Radiotherapy Breast cancer (LPRM). The LPRM defines evidence-based recommendations based on meetings with representatives of all Dutch radiotherapy institutions.

In the current study, tumours met the eligibility criteria for the FAST trial if they were: diagnosed in women ≥ 50 years, node-negative, <30 mm, treated with breast-conserving surgery resulting in negative resection margins, treated with whole-breast only radiotherapy without boost [10]. Tumours met the eligibility criteria for the FAST-Forward trial if they were: diagnosed in women ≥ 18 years, pTis-3, pN0-1, treated with surgery resulting in negative resection margins, not treated with regional radiotherapy [11]. These eligibility criteria differed slightly from the FAST and FAST-Forward trial, as patients with DCIS were not included in the trials, but were included in our study. Additionally, men were included in the FAST-Forward trial, but not in our study.

Hormone receptor positive (HR + ) was defined as oestrogen receptor (ER) and/or progesterone receptor (PR) positive tumours. Hormone receptor negative (HR-) was defined as ER and PR negative tumours. HR-status and HER2-status were combined in the variable ‘tumour subtype’ (HR+/HER2+, HR+/HER2−, HR−/HER2+, HR−/HER2 − ). Resection margins were classified as ‘negative’ if no invasive tumour and/or DCIS component was present in the inked margin, as ‘focally positive’ if the tumour component touched ≤ 4 mm of the inked margin, and as ‘extensively positive’ if the tumour component touched > 4 mm of the inked margin. An independent radiotherapy institution was defined as a radiotherapy institution not embedded in a hospital. A general and academic radiotherapy institution were defined as a radiotherapy institution embedded in a non-academic or academic hospital, respectively.

### 2.3 Statistics

Analyses were performed on tumour level instead of on patient level since women could have been included multiple times if they had subsequent breast tumours.

#### 2.3.1 Cohort A

Descriptive statistics were used to compare tumours included and not included in the NBCA-R. Chi-squared and Wilcoxon rank sum tests were used to compare groups. To investigate the coverage of the NBCA-R, the number and percentage of patients included and not included in the NBCA-R were shown for the three periods separately and by radiotherapy institution type.

#### 2.3.2 Cohort B

Descriptive statistics were used to report characteristics of all tumours, and stratified by type of radiotherapy schedule. Chi-squared and Wilcoxon rank sum tests were used to compare groups. Additionally, characteristics were described for tumours meeting the FAST and FAST-Forward criteria, stratified by type of radiotherapy schedule and period of diagnosis. The latter was performed to investigate whether characteristics of tumours receiving an ultra-hypofractionated schedule changed over time. Furthermore, for each month of 2020 and 2021 the percentage of tumours receiving a certain radiotherapy schedule was calculated to show trends over time. For this visual representation, the five fractions group was further divided in the five fractions of 5.2 Gy (FAST-Forward) and five fractions of 5.7 Gy (FAST) groups. Tumours with another five-fraction schedule were placed in the group ‘other’.

Logistic regression was used to calculate odds ratios (ORs) and 95 % confidence intervals (CIs) as a measure for the association between 1) age, 2) lateralization, 3) tumour grade, 4) tumour size, 5) pN, 6) neoadjuvant systemic treatment, 7) type of surgery, 8) PBI, and 9) adjuvant chemotherapy and the likelihood of receiving five fractions in tumours meeting the FAST- and FAST-Forward criteria. These analyses were performed for the first and second COVID-19 year separately.

All statistical analyses were performed in Stata Statistical Software (release 17.0, College Station, TX: StataCorp LLC). P-values were two-sided, and a p-value < 0.05 was regarded as statistically significant.

## 3 Results

**Fig. 1:**
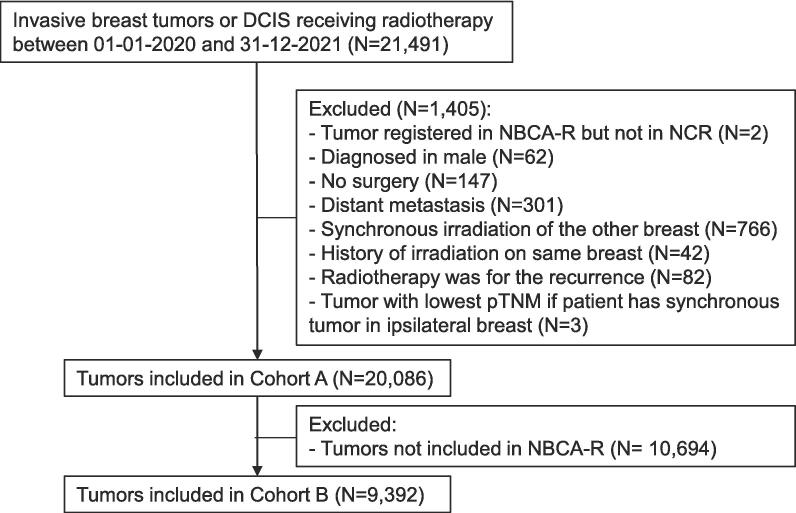
Flowchart of included tumours. NBCA-R: NABON Breast Cancer Audit-Radiotherapy, NCR: Netherlands Cancer Registry.

A total of 21,491 tumours, diagnosed in 21,074 patients, were eligible for inclusion. Of these tumours, 1,405 were excluded due to various reasons (Fig. 1). This resulted in 20,086 tumours, diagnosed in 20,067 patients, included in cohort A. A total of 10,694 tumours were not included in the NBCA-R, resulting in 9,392 tumours included in cohort B.

### 3.1 Cohort A

Characteristics of tumours included in the NBCA-R were approximately similar to those of the total group of irradiated tumours (Appendix Table A1). A total of 46.8 % of all tumours irradiated between 01–01-2020 and 31–12-2021 were included in the NBCA-R (Appendix Table A2). Tumours irradiated in independent radiotherapy institutions were underrepresented in the NBCA-R, with 40.6 % of the tumours irradiated in an independent radiotherapy institution being included in the NBCAR-R, compared to 49.3 % and 50.9 % of the tumours irradiated in a general or academic radiotherapy institution, respectively.

### 3.2 Cohort B

In total, 5,306 tumours received 15 fractions, 2,441 received 20 fractions, 1,523 received five fractions and 122 tumours received another radiotherapy schedule (Appendix Table A3). The detailed radiotherapy schedules of the four groups can be found in Appendix Table A4. Tumours meeting the criteria of the FAST or FAST-Forward trial more often received five fractions if they were diagnosed in older women and if they were < 30 mm and unifocal (Table 1, Table 2). Tumours meeting the criteria of the FAST-Forward trial more often received five fractions if they were grade 1 or 2, HR+/HER2-, <30 mm or pN0.

**Table 1:** Characteristics of tumours meeting the FAST criteria, in total and stratified by irradiation schedule and period of irradiation (N(%), unless otherwise specified).

	RT between 01–04-2020 and 31–12-2020				RT between 01–01-2021 and 31–12-2–21			
	**Total**	**Other**	**5 fractions**	**P-value**	**Total**	**Other**	**5 fractions**	**P-value**
Total	922	703	219		1,426	1,031	395	
Age				0.15				<0.001
50–75	820	631 (77.0)	189 (23.0)		1,328	976 (73.5)	352 (26.5)	
>75	102	72 (70.6)	30 (29.4)		98	55 (56.1)	43 (43.9)	
Diagnosed after screening				0.43				0.63
Yes	526	407 (77.4)	119 (22.6)		618	443 (71.7)	175 (28.3)	
No	390	293 (75.1)	97 (24.9)		806	587 (72.8)	219 (27.2)	
Unknown	6	3 (50.0)	3 (50.0)		2	1 (50.0)	1 (50.0)	
Lateralisation				0.029				0.17
Left	455	361 (79.3)	94 (20.7)		731	517 (70.7)	214 (29.3)	
Right	467	342 (73.2)	125 (26.8)		695	514 (74.0)	181 (26.0)	
Histology				0.59				0.29
Ductal	730	554 (75.9)	176 (24.1)		1,113	806 (72.4)	307 (27.6)	
Lobular/mixed	140	111 (79.3)	29 (20.7)		226	168 (74.3)	58 (25.7)	
Other	52	38 (73.1)	14 (26.9)		87	57 (65.5)	30 (34.5)	
Tumour grade				0.077				0.55
1 or 2	703	542 (77.1)	161 (22.9)		1,059	769 (72.6)	290 (27.4)	
3	189	134 (70.9)	55 (29.1)		341	242 (71.0)	99 (29.0)	
Unknown	30	27 (90.0)	3 (10.0)		26	20 (76.9)	6 (23.1)	
Subtype				0.32				0.004
HR+/HER2+	78	60 (76.9)	18 (23.1)		101	77 (76.2)	24 (23.8)	
HR+/HER2-	637	481 (75.5)	156 (24.5)		991	716 (72.3)	275 (27.7)	
HR-/HER2+	39	34 (87.2)	5 (12.8)		61	47 (77.0)	14 (23.0)	
HR-/HER2-	63	45 (71.4)	18 (28.6)		121	70 (57.9)	51 (42.1)	
Unknown/DCIS	105	83 (79.0)	22 (21.0)		152	121 (79.6)	31 (20.4)	
DCIS component				0.47				0.058
No	456	348 (76.3)	108 (23.7)		697	489 (70.2)	208 (29.8)	
Yes	372	279 (75.0)	93 (25.0)		589	432 (73.3)	157 (26.7)	
DCIS	90	73 (81.1)	17 (18.9)		138	110 (79.7)	28 (20.3)	
Unknown	4	3 (75.0)	1 (25.0)		2	0 (0.0)	2 (100.0)	
Tumour size (mm)				0.25				0.041
pTis	90	73 (81.1)	17 (18.9)		138	110 (79.7)	28 (20.3)	
<30	832	630 (75.7)	202 (24.3)		1,288	921 (71.5)	367 (28.5)	
Multifocality				0.53				0.006
No	832	633 (76.1)	199 (23.9)		1,297	924 (71.2)	373 (28.8)	
Yes	86	68 (79.1)	18 (20.9)		127	105 (82.7)	22 (17.3)	
Unknown	4	2 (50.0)	2 (50.0)		2	2 (100.0)	0 (0.0)	
Neo-adjuvant therapy				0.94				0.25
No	751	573 (76.3)	178 (23.7)		1,175	857 (72.9)	318 (27.1)	
Yes	171	130 (76.0)	41 (24.0)		251	174 (69.3)	77 (30.7)	
Adjuvant chemotherapy				0.69				0.14
No	856	654 (76.4)	202 (23.6)		1,326	965 (72.8)	361 (27.2)	
Yes	66	49 (74.2)	17 (25.8)		100	66 (66.0)	34 (34.0)	
Adjuvant endocrine therapy				0.056				0.003
No	577	428 (74.2)	149 (25.8)		905	630 (69.6)	275 (30.4)	
Yes	345	275 (79.7)	70 (20.3)		521	401 (77.0)	120 (23.0)	
Adjuvant targeted therapy				0.57				0.19
No	824	626 (76.0)	198 (24.0)		1,287	924 (71.8)	363 (28.2)	
Yes	98	77 (78.6)	21 (21.4)		139	107 (77.0)	32 (23.0)	
Type of RT institution				<0.001				<0.001
Independent	368	313 (85.1)	55 (14.9)		466	420 (90.1)	46 (9.9)	
General	154	133 (86.4)	21 (13.6)		266	211 (79.3)	55 (20.7)	
Academic	400	257 (64.3)	143 (35.8)		694	400 (57.6)	294 (42.4)	
Travel time by car (min) Median (IQR)	19.0 (13.0–26.0)	19.0 (13.0–26.0)	20.0 (12.0–27.0)	0.54	21.0 (14.0–27.0)	21.0 (15.0–27.0)	20.0 (12.0–28.0)	0.067

HR: Hormone receptor, IQR: interquartile range, RT: radiotherapy.

**Table 2:** Characteristics of tumours meeting the FAST-Forward criteria, in total and stratified by irradiation schedule and period of irradiation (N(%), unless otherwise specified).

	RT between 01–04-2020 and 31–12-2020				RT between 01–01-2021 and 31–12-2–21			
	**Total**	**Other**	**5 fractions**	**P-value**	**Total**	**Other**	**5 fractions**	**P-value**
Total	2,115	1,711	404		3,137	2,247	890	
Age				<0.001				<0.001
<50	394	357 (90.6)	37 (9.4)		497	429 (86.3)	68 (13.7)	
50–75	1,553	1,225 (78.9)	328 (21.1)		2,424	1,690 (69.7)	734 (30.3)	
>75	168	129 (76.8)	39 (23.2)		216	128 (59.3)	88 (40.7)	
Diagnosed after screening				<0.001				<0.001
Yes	1,358	1,138 (83.8)	220 (16.2)		1,595	1,217 (76.3)	378 (23.7)	
No	750	569 (75.9)	181 (24.1)		1,537	1,026 (66.8)	511 (33.2)	
Unknown	7	4 (57.1)	3 (42.9)		5	4 (80.0)	1 (20.0)	
Lateralisation				0.048				0.027
Left	1,080	892 (82.6)	188 (17.4)		1,576	1,101 (69.9)	475 (30.1)	
Right	1,035	819 (79.1)	216 (20.9)		1,561	1,146 (73.4)	415 (26.6)	
Histology				0.51				0.10
Ductal	1,746	1,410 (80.8)	336 (19.2)		2,603	1,856 (71.3)	747 (28.7)	
Lobular	228	190 (83.3)	38 (16.7)		360	273 (75.8)	87 (24.2)	
Other	141	111 (78.7)	30 (21.3)		174	118 (67.8)	56 (32.2)	
Tumour grade				<0.001				<0.001
1 or 2	1,438	1,117 (77.7)	321 (22.3)		2,207	1,466 (66.4)	741 (33.6)	
3	616	537 (87.2)	79 (12.8)		869	729 (83.9)	140 (16.1)	
Unknown	61	57 (93.4)	4 (6.6)		61	52 (85.2)	9 (14.8)	
Subtype				<0.001				<0.001
HR+/HER2+	165	140 (84.8)	25 (15.2)		219	185 (84.5)	34 (15.5)	
HR+/HER2-	1,361	1,058 (77.7)	303 (22.3)		2,095	1,394 (66.5)	701 (33.5)	
HR-/HER2+	82	75 (91.5)	7 (8.5)		122	101 (82.8)	21 (17.2)	
HR-/HER2-	276	248 (89.9)	28 (10.1)		352	285 (81.0)	67 (19.0)	
Unknown/DCIS	231	190 (82.3)	41 (17.7)		349	282 (80.8)	67 (19.2)	
DCIS component				0.66				<0.001
No	1,024	822 (80.3)	202 (19.7)		1,482	1,033 (69.7)	449 (30.3)	
Yes	881	715 (81.2)	166 (18.8)		1,334	957 (71.7)	377 (28.3)	
DCIS	205	170 (82.9)	35 (17.1)		318	256 (80.5)	62 (19.5)	
Unknown	5	4 (80.0)	1 (20.0)		3	1 (33.3)	2 (66.7)	
Tumour size (mm)				0.14				<0.001
pTis	227	190 (83.7)	37 (16.3)		353	289 (81.9)	64 (18.1)	
<30	1,762	1,413 (80.2)	349 (19.8)		2,615	1,808 (69.1)	807 (30.9)	
>30	117	101 (86.3)	16 (13.7)		153	136 (88.9)	17 (11.1)	
Missing	9	7 (77.8)	2 (22.2)		16	14 (87.5)	2 (12.5)	
pN/ypN				0.006				<0.001
0	1,965	1,577 (80.3)	388 (19.7)		2,958	2,087 (70.6)	871 (29.4)	
1	150	134 (89.3)	16 (10.7)		179	160 (89.4)	19 (10.6)	
Multifocality				0.021				<0.001
No	1,897	1,523 (80.3)	374 (19.7)		2,877	2,023 (70.3)	854 (29.7)	
Yes	213	185 (86.9)	28 (13.1)		254	218 (85.8)	36 (14.2)	
Unknown	5	3 (60.0)	2 (40.0)		6	6 (100.0)	0 (0.0)	
Neo-adjuvant therapy				<0.001				<0.001
No	1,626	1,283 (78.9)	343 (21.1)		2,487	1,710 (68.8)	777 (31.2)	
Yes	489	428 (87.5)	61 (12.5)		650	537 (82.6)	113 (17.4)	
Surgery				0.13				<0.001
Breast conserving therapy	2,029	1,636 (80.6)	393 (19.4)		3,052	2,172 (71.2)	880 (28.8)	
Mastectomy	86	75 (87.2)	11 (12.8)		85	75 (88.2)	10 (11.8)	
Direct reconstruction				0.12				0.016
No	2,096	1,693 (80.8)	403 (19.2)		3,116	2,227 (71.5)	889 (28.5)	
Yes	19	18 (94.7)	1 (5.3)		21	20 (95.2)	1 (50.0)	
Adjuvant chemotherapy				<0.001				<0.001
No	1,804	1,433 (79.4)	371 (20.6)		2,754	1,912 (69.4)	842 (30.6)	
Yes	311	278 (89.4)	33 (10.6)		383	335 (87.5)	48 (12.5)	
Adjuvant endocrine therapy				<0.001				<0.001
No	1,269	994 (78.3)	275 (21.7)		1,959	1,318 (67.3)	641 (32.7)	
Yes	846	717 (84.8)	129 (15.2)		1,178	929 (78.9)	249 (21.1)	
Adjuvant targeted therapy				0.034				<0.001
No	1,909	1,533 (80.3)	376 (19.7)		2,846	1,998 (70.2)	848 (29.8)	
Yes	206	178 (86.4)	28 (13.6)		291	249 (85.6)	42 (14.4)	
Partial Breast Irradiation				<0.001				<0.001
No	1,880	1,593 (84.7)	287 (15.3)		2,578	2,081 (80.7)	497 (19.3)	
Yes	232	116 (50.0)	116 (50.0)		557	165 (29.6)	392 (70.4)	
Missing	3	2 (66.7)	1 (33.3)		2	1 (50.0)	1 (50.0)	
Type of RT institution				<0.001				<0.001
Independent	759	664 (87.5)	95 (12.5)		966	839 (86.9)	127 (13.1)	
General	373	345 (92.5)	28 (7.5)		650	495 (76.2)	155 (23.8)	
Academic	983	702 (71.4)	281 (28.6)		1,521	913 (60.0)	608 (40.0)	
Travel time by car (min) Median (IQR)	19.0 (13.0–26.0)	19.0 (13.0–26.0)	19.0 (12.0–27.0)	0.96	21.0 (14.0–27.0)	21.0 (14.0–27.0)	19.0 (13.0–27.0)	0.005

HR: Hormone receptor, IQR: interquartile range, RT: radiotherapy.

**Fig. 2:**
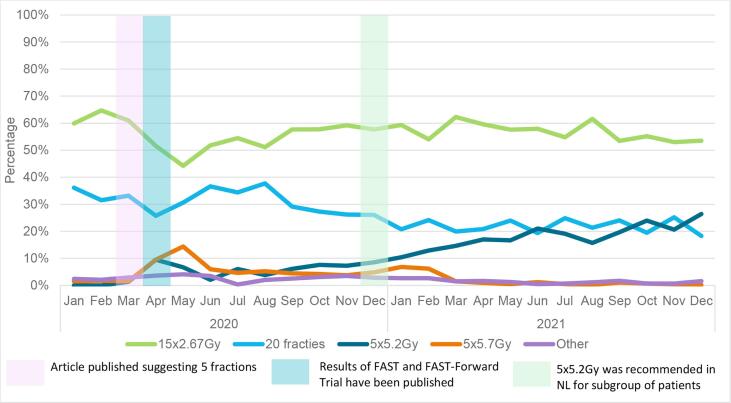

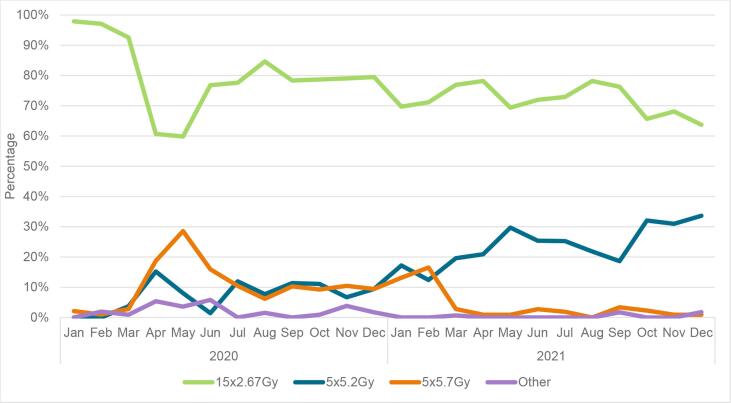

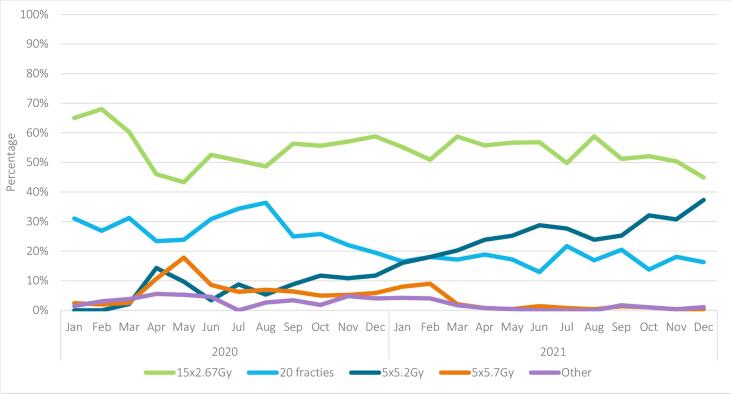
Radiotherapy schedule of patients treated with radiotherapy in the Netherlands (NL) over the months of radiotherapy treatment for (A) all tumours treated with radiotherapy, (B) tumours meeting the FAST criteria, (C) tumours meeting the FAST-Forward criteria.

The 5x5.2 Gy schedule was first implemented in March 2020, and 10 % of the irradiated tumours received this schedule in April 2020 (Fig. 2A). The use of this schedule remained relatively constant between May–December 2020, and increased thereafter. By December 2021, 26 % of the tumours were treated with 5x5.2 Gy. Usage of the 5x5.7 Gy schedule peaked at 10 % and 14 % in April and May 2020, respectively, after which it stayed relatively constant. From March 2021 onwards the use of this schedule decreased, with 0.2 % of the tumours receiving 5x5.7 Gy in December 2021. Fig. 2B and 2C shows the usage of the radiotherapy schedules in tumours meeting the FAST and FAST-Forward criteria.

Multivariable logistic regression was performed for tumours meeting the FAST criteria. For tumours irradiated in 2020, lateralization and tumour grade were significantly associated with the administration of five fractions (Table 3). In 2021, age was significantly associated with the administration of five fractions. In both 2020 and 2021, there was a significant association with the type of radiotherapy institution.

**Table 3:** Association between patient-, tumour-, treatment- and radiotherapy institution-related characteristics and likelihood of receiving five fractions in tumours meeting the FAST criteria, stratified by period of radiotherapy.

	RT between 01–04-2020 and 31–12-2020		RT between 01–01-2021 and 31–12-2–21	
	Crude OR (95 % CI)^a^ (N = 922)	Adjusted HR (95 % CI) (N = 892)	Crude OR (95 % CI)^a^ (N = 1,426)	Adjusted HR (95 % CI) (N = 1,398)
Age				
50–75	1.00 (reference)	1.00 (reference)	1.00 (reference)	1.00 (reference)
>75	1.39 (0.88–2.19)	1.54 (0.94–2.52)	2.17 (1.43–3.29)	2.47 (1.55–3.91)
Lateralisation				
Left	1.00 (reference)	1.00 (reference)	1.00 (reference)	1.00 (reference)
Right	1.40 (1.03–1.91)	1.48 (1.07–2.04)	0.85 (0.67–1.07)	0.84 (0.65–1.08)
Tumour grade				
1 or 2	1.00 (reference)	1.00 (reference)	1.00 (reference)	1.00 (reference)
3	1.38 (0.96–1.98)	1.57 (1.02–2.42)	1.08 (0.83–1.42)	1.15 (0.82–1.62)
Tumour size				
pTis	0.73 (0.42–1.26)	0.63 (0.33–1.21)	0.64 (0.41–0.98)	0.83 (0.50–1.38)
<30	1.00 (reference)	1.00 (reference)	1.00 (reference)	1.00 (reference)
Neoadjuvant therapy				
No	1.00 (reference)	1.00 (reference)	1.00 (reference)	1.00 (reference)
Yes	1.02 (0.69–1.50)	1.06 (0.67–1.67)	1.19 (0.89–1.61)	1.33 (0.92–1.91)
Adjuvant chemotherapy				
No	1.00 (reference)	1.00 (reference)	1.00 (reference)	1.00 (reference)
Yes	1.12 (0.63–1.99)	1.14 (0.60–2.14)	1.38 (0.89–2.12)	1.27 (0.78–2.07)
Type of RT institution				
Independent	1.00 (reference)	1.00 (reference)	1.00 (reference)	1.00 (reference)
General	0.90 (0.42–1.55)	0.87 (0.50–1.51)	2.38 (1.56–3.64)	2.30 (1.49–3.55)
Academic	3.17 (2.23–4.50)	3.33 (2.31–4.80)	6.71 (4.78–9.42)	6.70 (4.73–9.47)
Travel time by car (per 10 min)	1.03 (0.90–1.18)	1.13 (0.97–1.32)	0.90 (0.81–1.00)	0.98 (0.87–1.10)

CI: confidence interval, HR: Hormone receptor, OR: Odds ratio, RT: radiotherapy.

^a^Number of patients accounts for the analyses concerning variables without missing data.

For tumours meeting the FAST-Forward criteria, and irradiated in 2020, lateralization was significantly associated with the administration of five fractions (Table 4). For tumours irradiated in 2020 or 2021, age was significantly associated with the administration of five fractions, as was the pN/ypN, PBI, and the type of radiotherapy institution. In 2021, there was a significant association with pT/tumour size.

**Table 4:** Association between patient-, tumour-, treatment- and radiotherapy institution-related characteristics and likelihood of receiving five fractions in tumours meeting the FAST-Forward criteria, stratified by period of radiotherapy.

	RT between 01–04-2020 and 31–12-2020		RT between 01–01-2021 and 31–12-2–21	
	Crude OR (95 % CI)^a^ (N = 2,115)	Adjusted HR (95 % CI) (N = 2,042)	Crude OR (95 % CI)^a^ (N = 3,137)	Adjusted HR (95 % CI) (N = 3,056)
Age				
<50	1.00 (reference)	1.00 (reference)	1.00 (reference)	1.00 (reference)
50–75	2.58 (1.80–3.70)	2.11 (1.44–3.11)	2.74 (2.09–3.59)	1.86 (1.37–2.54)
>75	2.92 (1.78–4.77)	3.21 (1.86–5.54)	4.34 (2.99–6.30)	4.23 (2.71–6.61)
Lateralisation				
Left	1.00 (reference)	1.00 (reference)	1.00 (reference)	1.00 (reference)
Right	1.25 (1.01–1.56)	1.42 (1.11–1.80)	0.84 (0.72–0.98)	0.84 (0.70–1.02)
Tumour grade				
1 or 2	1.00 (reference)	1.00 (reference)	1.00 (reference)	1.00 (reference)
3	0.51 (0.39–0.67)	0.74 (0.54–1.02)	0.38 (0.31–0.46)	0.86 (0.66–1.11)
Tumour size				
pTis	0.79 (0.54–1.14)	0.99 (0.65–1.53)	0.50 (0.37–0.66)	0.58 (0.41–0.81)
<30 mm	1.00 (reference)	1.00 (reference)	1.00 (reference)	1.00 (reference)
>30 mm	0.64 (0.37–1.10)	0.92 (0.49–1.73)	0.28 (0.17–0.47)	0.42 (0.24–0.74)
pN/ypN				
0	1.00 (reference)	1.00 (reference)	1.00 (reference)	1.00 (reference)
1	0.49 (0.29–0.82)	0.55 (0.30–0.98)	0.28 (0.18–0.46)	0.43 (0.26–0.73)
Neoadjuvant therapy				
No	1.00 (reference)	1.00 (reference)	1.00 (reference)	1.00 (reference)
Yes	0.53 (0.40–0.71)	0.90 (0.64–1.26)	0.46 (0.37–0.58)	1.00 (0.76–1.31)
Surgery				
Breast conserving surgery	1.00 (reference)	1.00 (reference)	1.00 (reference)	1.00 (reference)
Mastectomy	0.61 (0.32–1.16)	1.21 (0.55–2.66)	0.33 (0.17–0.64)	0.82 (0.36–1.83)
Adjuvant chemotherapy				
No	1.00 (reference)	1.00 (reference)	1.00 (reference)	1.00 (reference)
Yes	0.46 (0.31–0.67)	0.78 (0.51–1.20)	0.33 (0.24–0.45)	0.60 (0.42–0.85)
Partial Breast Irradiation				
No	1.00 (reference)	1.00 (reference)	1.00 (reference)	1.00 (reference)
Yes	5.55 (4.17–7.39)	4.36 (3.14–6.07)	9.95 (8.09–12.23)	9.15 (7.17–11.68)
Type of RT institution				
Independent	1.00 (reference)	1.00 (reference)	1.00 (reference)	1.00 (reference)
General	0.57 (0.36–0.88)	0.52 (0.33–0.82)	2.07 (1.60–2.68)	1.66 (1.23–2.24)
Academic	2.80 (2.17–3.61)	2.98 (2.26–3.92)	4.40 (3.56–5.44)	5.35 (4.17–6.86)
Travel time by car (per 10 min)	0.99 (0.90–1.09)	1.08 (0.97–1.20)	0.90 (0.84–0.97)	0.97 (0.89–1.06)

CI: confidence interval, HR: Hormone receptor, OR: Odds ratio, RT: radiotherapy.

^a^Number of patients accounts for the analyses concerning variables without missing data.

## 4 Discussion

The current study showed the trends in the use of different radiotherapy schedules in breast cancer patients during the COVID-19 pandemic. Additionally, we showed that tumours meeting the criteria of the FAST-Forward trial were more likely to be treated with five fractions if they received PBI compared to whole breast irradiation, or when they were irradiated at an academic compared to an independent radiotherapy institution.

Our results showed that the 26 Gy schedule was first used in March 2020. This was together with the start of the COVID-19 pandemic in the Netherlands and the national and international suggestion to use ultra-hypofractionated radiotherapy schedules for a selected group of patients [13], and one month before the five-year results of the FAST-Forward trial were actually published. The usage of the 28.5 Gy schedule peaked in April and May 2020, while the 10-year results of the FAST trial were published in July 2020. The international suggestion to use ultra-hypofractionated radiotherapy schedules was co-authored by researchers of the FAST and FAST-Forward trial. The knowledge of these researchers, together with previous published results on these trials [14], [15], [16], probably formed the basis for the recommendation. One of the authors of the national recommendation also co-authored the international recommendation. Knowledge gained via personal communication during the writing of the international recommendation, still in press at the time the national recommendation was published, was used when writing the national recommendation. This first version of the national recommendation was only meant to be used during personnel shortages due to the pandemic. A further increase in the use of the 26 Gy schedule was seen after the National Platform Radiotherapy Breast cancer (LPRM) recommended this schedule for a selected group of patients (see Material and Methods). Usually, it takes more time before new recommendations and treatments are formed and implemented. This study thereby shows the accelerating role of the COVID-19 pandemic and the national radiotherapy society. We therefore highly recommended the formation of national radiotherapy societies, which take the lead in incorporating new scientific data into national guidelines, to stimulate the implementation of new treatments. A decrease in the use of the 28.5 Gy schedule was seen in 2021. This decrease could be due to fear of toxicity of the 28.5 Gy schedule [10], [18], the more preferred endpoint (i.e. risk of recurrence) and the larger sample size of the FAST-Forward trial compared to the FAST trial, the recommendation to use the 26 Gy schedule by the LPRM, and the shorter duration of treatment with the 26 Gy schedule.

In December 2021, 38 % of the irradiated tumours meeting the Fast-Forward trial criteria received 26 Gy. Even though the results of the FAST-Forward study showed no increase in the risk of normal tissue effects for the 26 Gy schedule, the hesitance to use this schedule could be due to fear of toxicity, as only the five-year results of this study are available [11]. This fear has no basis in literature, since the START-B trial and FAST trial showed that the hazard ratio for late normal tissue effects tends to be similar after five and 10 years of follow-up [10], [19]. Another possible reason for the reluctance to use the 26 Gy schedule could be that in the Netherlands, a higher reimbursement can be declared for women receiving left-sided radiotherapy in combination with a breath-hold technique, provided they receive at least six fractions. This is supported by the finding of the current study that left-sided tumours irradiated in 2020 were treated less often with a five-fraction schedule.

Tumours meeting the criteria for the FAST or FAST-Forward trial were more likely to receive five fractions if they were diagnosed in older women. Institutions might be waiting for the 10-year results before implementing this schedule in relatively young patients. Subgroup analyses, published in 2021, comparing 26 Gy with 40 Gy showed no evidence of a difference in recurrence risk or moderate/marked normal tissue effects according to age [20]. Additionally, it is possible that during the pandemic, radiotherapy institutions especially wanted to reduce the number of hospital visits in elderly patients. Furthermore, our results showed that tumours meeting the criteria for the FAST-Forward trial were more likely to receive five fractions if they were treated with PBI. Tumours of > 30 mm or pN1 tumours were less likely to receive five fractions. This indicates that radiation oncologist were more likely to use five fractions in tumours with a low risk of recurrence [21], [22]. In addition, radiation oncologist probably expected a lower toxicity risk when using PBI, because a smaller volume is than irradiated. Patients receiving regional radiotherapy were not included in the logistic analyses of this study. These patients were treated in the FAST-Forward nodal Trial, that is currently in the follow-up phase. Finally, the lower usage of the five-fraction schedule in independent radiotherapy institutions could be due to the reduced pressure of the COVID-19 pandemic in these institutions, as they are not part of an hospital. In general and academic radiotherapy institutions, staff could have been relocated to other healthcare departments, which might have resulted in shortages at the radiotherapy-department. To deal with this the ultra-hypofractionated schedule might have been more likely to be implemented.

Guidelines have been updated increasingly since the publication of the FAST and FAST-Forward trial. For instance, both the NCCN and ESTRO now endorse ultra-hypofractionations as a standard choice for selected patients [23], [24]. A study from the United Kingdom (UK) showed that 80 % of the women > 50 years, and diagnosed with a stage 0–IIIA tumour, received 26 Gy at the end of April 2020 [25]. A single institution study performed in Mumbai, India, showed that the average number of fractions received by breast cancer patients dropped from 14.4 in March–October 2019 to 6.2 in March–October 2020 [26]. The quick implementation of this schedule in these countries could be because of the known shortage in radiotherapy capacity in both countries. Additionally, the high percentage found in the UK could be because the FAST and FAST-Forward trial were both located in the UK.

Strengths of this study include the large number of variables included in the current study. A limitation is that only a selection of the radiotherapy institutions were included. Independent radiotherapy institutions are underrepresented, with 47.8 % and 38.5 % of the patients irradiated in independent radiotherapy institutions being included in the 2020 and 2021 data of the NBCA-R, respectively. General and academic radiotherapy institutions are overrepresented. Independent radiotherapy institutions less often used the five-fraction schedules, so the results might be a little too optimistic, especially for 2021. In addition, the NBCA-R covered only 32.8 % of the irradiated patients in the pre-COVID period, as the NBCA-R only started in 2020. However, as the five-fraction schedules were barely used during this period, we do not expect that this has influenced the results. The lower degree of implementation of the five-fraction schedules in independent institutes could be due to the fact that these areas were less affected by the pandemic, or due to financial issues. Importantly, the NBCA-R started in 2020 and therefore included not all treated patients from the registering radiotherapy institutions. Lastly, some of the patient groups included a small number of patients, thereby limiting the power of the analyses involving those patients.

## 5 Conclusions

Our results showed that at the start of the COVID-19 pandemic, the use of the 28 Gy schedule increased fivefold and the 26 Gy schedule was used for the first time in a selected group of patients. A steep increase in the use of the 26 Gy schedule was seen after its use was recommended by the National Platform Radiotherapy Breast cancer (LPRM). It seems that recommendations of a national body stimulate implementation of a new treatment (technique). We therefore recommend the formation of national radiotherapy societies, which take the lead in incorporating new scientific data into national guidelines, to stimulate and accelerate the implementation of new treatments. Since ultra-hypofractionation is not yet used for all eligible patients, other drivers of change need to be found.

## CRediT authorship contribution statement

**Anouk H. Eijkelboom:** Conceptualization, Data curation, Formal analysis, Investigation, Methodology, Project administration, Resources, Visualization, Writing – original draft, Writing – review & editing. **Marcel R. Stam:** Conceptualization, Resources, Writing – review & editing. **Desirée H.J.G. van den Bongard:** Conceptualization, Resources, Writing – review & editing. **Margriet G.A. Sattler:** Resources, Writing – review & editing. **Enja J. Bantema-Joppe:** Resources, Writing – review & editing. **Sabine Siesling:** Conceptualization, Funding acquisition, Writing – review & editing. **Marissa C. van Maaren:** Conceptualization, Investigation, Project administration, Resources, Writing – review & editing.

## Declaration of competing interest

The authors declare that they have no known competing financial interests or personal relationships that could have appeared to influence the work reported in this paper.
