# Supplementary material for: Implementation of ultra-hypofractionated radiotherapy schedules for breast cancer during the COVID-19 pandemic in the Netherlands
Source: Clin Transl Radiat Oncol. 2024 Jun 14;47:100807. doi: 10.1016/j.ctro.2024.100807 (PMC11228630; doi:10.1016/j.ctro.2024.100807)
Supplement: Supplementary Data 1 [file mmc1.docx]

**Table A.1** Characteristics of women included and not included in the NBCA-R, (N(%)).

|  | Total | Not included in NBCA-R | Included in NBCA-R | P-value |
| --- | --- | --- | --- | --- |
| Total | 20,086 | 10,694 | 9,392 |  |
| Age |  |  |  | <0.001 |
| <50 | 4,036 (20.1) | 2,272 (21.2) | 1,764 (18.8) |  |
| 50-75 | 14,332 (71.4) | 7,529 (70.4) | 6,803 (72.4) |  |
| >75 | 1,718 (8.6) | 893 (8.4) | 825 (8.8) |  |
| Diagnosed after screening |  |  |  | 0.24 |
| Yes | 12,170 (60.6) | 6,370 (59.6) | 5,800 (61.8) |  |
| No | 7,338 (36.5) | 3,777 (35.3) | 3,561 (37.9) |  |
| Unknown | 578 (2.9) | 547 (5.1) | 31 (0.3) |  |
| Lateralisation |  |  |  | 0.017 |
| Left | 10,212 (50.8) | 5,521 (51.6) | 4,691 (49.9) |  |
| Right | 9,874 (49.2) | 5,173 (48.4) | 4,701 (50.1) |  |
| Histology |  |  |  | 0.31 |
| Ductal | 16,250 (80.9) | 8,653 (80.9) | 7,597 (80.9) |  |
| Lobular/mixed | 2,791 (13.9) | 1,506 (14.1) | 1,285 (13.7) |  |
| Other | 1,045 (5.2) | 535 (5.0) | 510 (5.4) |  |
| Tumour grade |  |  |  | 0.34 |
| 1 or 2 | 14,009 (69.7) | 7,499 (70.1) | 6,510 (69.3) |  |
| 3 | 5,555 (27.7) | 2,932 (27.4) | 2,623 (27.9) |  |
| Unknown | 522 (2.6) | 263 (2.5) | 259 (2.8) |  |
| Subtype |  |  |  | 0.12 |
| HR+/HER2+ | 1,619 (8.1) | 907 (8.5) | 712 (7.6) |  |
| HR+/HER2- | 13,819 (68.8) | 7,400 (69.2) | 6,419 (68.3) |  |
| HR-/HER2+ | 755 (3.8) | 388 (3.6) | 367 (3.9) |  |
| HR-/HER2- | 2,201 (11.0) | 1,163 (10.9) | 1,038 (11.1) |  |
| Unknown/DCIS | 1,692 (8.4) | 836 (7.8) | 856 (9.1) |  |
| DCIS component |  |  |  | <0.001 |
| No | 9,871 (49.1) | 5,424 (50.7) | 4,447 (47.3) |  |
| Yes | 8,217 (40.9) | 4,007 (37.5) | 4,210 (44.8) |  |
| DCIS | 1,472 (7.3) | 755 (7.1) | 717 (7.6) |  |
| Unknown | 526 (2.6) | 508 (4.8) | 18 (0.2) |  |
| Tumour size (mm) |  |  |  | 0.03 |
| pTis | 1,721 (8.6) | 880 (8.2) | 841 (9.0) |  |
| <30 | 15,857 (78.9) | 8,444 (79.0) | 7,413 (78.9) |  |
| >30 | 2,101 (10.5) | 1,163 (10.9) | 938 (10.0) |  |
| pT4 | 113 (0.6) | 68 (0.6) | 45 (0.5) |  |
| Unknown | 239 (1.2) | 116 (1.1) | 123 (1.3) |  |
| pN/ypN |  |  |  | 0.002 |
| 0 | 13,244 (65.9) | 6,885 (64.4) | 6,359 (67.7) |  |
| 1 | 4,957 (24.7) | 2,731 (25.5) | 2,226 (23.7) |  |
| 2 | 558 (2.8) | 303 (2.8) | 255 (2.7) |  |
| 3 | 268 (1.3) | 149 (1.4) | 119 (1.3) |  |
| Unknown | 1,059 (5.3) | 626 (5.9) | 433 (4.6) |  |
| Multifocality |  |  |  | 0.004 |
| No | 16,919 (84.2) | 8,926 (83.5) | 7,993 (85.1) |  |
| Yes | 3,078 (15.3) | 1,710 (16.0) | 1,368 (14.6) |  |
| Unknown | 89 (0.4) | 58 (0.5) | 31 (0.3) |  |
| Neo-adjuvant therapy |  |  |  | 0.001 |
| No | 13,865 (69.0) | 7,276 (68.0) | 6,589 (70.2) |  |
| Yes | 6,221 (31.0) | 3,418 (32.0) | 2,803 (29.8) |  |
| Surgery |  |  |  | <0.001 |
| Breast conserving therapy | 16,749 (83.4) | 8,762 (81.9) | 7,987 (85.0) |  |
| Mastectomy | 3,308 (16.5) | 1,911 (17.9) | 1,397 (14.9) |  |
| Unknown | 29 (0.1) | 21 (0.2) | 8 (0.1) |  |
| Direct reconstruction |  |  |  | <0.001 |
| No | 19,217 (95.7) | 10,124 (94.7) | 9,093 (96.8) |  |
| Yes | 869 (4.3) | 570 (5.3) | 299 (3.2) |  |
| Resection margins |  |  |  | 0.10 |
| Negative | 18,094 (90.1) | 9,540 (89.2) | 8,554 (91.1) |  |
| Focally positive | 1,580 (7.9) | 875 (8.2) | 705 (7.5) |  |
| Extensively positive | 138 (0.7) | 77 (0.7) | 61 (0.6) |  |
| Unknown | 274 (1.4) | 202 (1.9) | 72 (0.8) |  |
| Adjuvant chemotherapy |  |  |  | 0.078 |
| No | 16,776 (83.5) | 8,978 (84.0) | 7,798 (83.0) |  |
| Yes | 3,310 (16.5) | 1,716 (16.0) | 1,594 (17.0) |  |
| Adjuvant endocrine therapy |  |  |  | <0.001 |
| No | 9,966 (49.6) | 5,141 (48.1) | 4,825 (51.4) |  |
| Yes | 10,120 (50.4) | 5,553 (51.9) | 4,567 (48.6) |  |
| Adjuvant targeted therapy |  |  |  | 0.026 |
| No | 17,981 (89.5) | 9,525 (89.1) | 8,456 (90.0) |  |
| Yes | 2,105 (10.5) | 1,169 (10.9) | 936 (10.0) |  |
| Irradiation of lymph nodes |  |  |  | <0.001 |
| No | 13,867 (69.0) | 7,176 (67.1) | 6,691 (71.2) |  |
| Yes | 5,792 (28.8) | 3,261 (30.5) | 2,531 (26.9) |  |
| Missing | 427 (2.1) | 257 (2.4) | 170 (1.8) |  |
| Partial Breast Irradiation |  |  |  | <0.001 |
| No | 18,129 (90.3) | 9,673 (90.5) | 8,456 (90.0) |  |
| Yes | 1,654 (8.2) | 805 (7.5) | 849 (9.0) |  |
| Missing | 303 (1.5) | 216 (2.0) | 87 (0.9) |  |
| HR: Hormone receptor, NBCA-R: Netherlands Breast Cancer Audit – Radiotherapy | | | | |

**Table A.2** Coverage of the NBCA-R compared to the NCR, N(%).

|  | Total of irradiated patients in the NCR | | Pre-COVID period | | 2020 | | 2021 | |
| --- | --- | --- | --- | --- | --- | --- | --- | --- |
|  | **Not included in NBCA-R** | **Included in NBCA-R** | **Not included in NBCA-R** | **Included in NBCA-R** | **Not included in NBCA-R** | **Included in NBCA-R** | **Not included in NBCA-R** | **Included in NBCA-R** |
| Total | 10,694 (53.2) | 9,392 (46.8) | 1,555 (67.2) | 760 (32.8) | 3,399 (48.7) | 3,584 (51.3) | 5,740 (53.2) | 5,048 (46.8) |
| NBCA-R institution^a^ |  |  |  |  |  |  |  |  |
| Yes | 3,150 (25.1) | 9,392 (74.9) | 676  (47.1) | 760 (52.9) | 806  (18.4) | 3,584 (81.6) | 1,668 (24.8) | 5,048 (75.2) |
| Type of RT institution |  |  |  |  |  |  |  |  |
| Independent | 4,471 (59.4) | 3,055 (40.6) | 614  (71.4) | 246 (28.6) | 1,350 (52.2) | 1,238 (47.8) | 2,507 (61.5) | 1,571 (38.5) |
| General | 1,860 (50.7) | 1,806 (49.3) | 247  (59.2) | 170 (40.8) | 666  (51.4) | 630 (48.6) | 947  (48.5) | 1,006 (51.5) |
| Academic | 4,363 (49.1) | 4,531 (50.9) | 694  (66.9) | 344 (33.1) | 1,383 (44.6) | 1,716 (55.4) | 2,286 (48.1) | 2,471 (51.9) |
| NBCA-R: NABON Breast Cancer Audit-Radiotherapy; NCR: Netherlands Cancer Registry   1. A radiotherapy centre which provided radiotherapy data to the NBCA-R | | | | | | | | |

**Table A.3** Baseline characteristics in total and stratified by irradiation schedule (N(%), unless otherwise specified).

|  | Total | 15 fractions | 20 fractions | 5 fractions | Other | P-value |
| --- | --- | --- | --- | --- | --- | --- |
| Total | 9,392 | 5,306 | 2,441 | 1,523 | 122 |  |
| Period of RT |  |  |  |  |  | <0.001 |
| Pre-COVID | 760 (8.1) | 463 (8.7) | 271 (11.1) | 11 (0.7) | 15 (12.3) |  |
| First COVID-year | 3,584 (38.2) | 1,967 (37.1) | 1,065 (43.6) | 493 (32.4) | 59 (48.4) |  |
| Second COVID-year | 5,048 (53.7) | 2,876 (54.2) | 1,105 (45.3) | 1,019 (66.9) | 48 (39.3) |  |
| Age |  |  |  |  |  | <0.001 |
| <50 | 1,764 (18.8) | 888 (16.7) | 744 (30.5) | 116 (7.6) | 16 (13.1) |  |
| 50-75 | 6,803 (72.4) | 3,920 (73.9) | 1,556 (63.7) | 1,238 (81.3) | 89 (73.0) |  |
| >75 | 825 (8.8) | 498 (9.4) | 141 (5.8) | 169 (11.1) | 17 (13.9) |  |
| Diagnosed after screening |  |  |  |  |  | <0.001 |
| Yes | 5,800 (61.8) | 3,360 (63.3) | 1,643 (67.3) | 723 (47.5) | 74 (60.7) |  |
| No | 3,561 (37.9) | 1,926 (36.3) | 794 (32.5) | 793 (52.1) | 48 (39.3) |  |
| Unknown | 31 (0.3) | 20 (0.4) | 4 (0.2) | 7 (0.5) | 0 (0.0) |  |
| Lateralisation |  |  |  |  |  | 0.24 |
| Left | 4,691 (49.9) | 2,625 (49.5) | 1,223 (50.1) | 772 (50.7) | 71 (58.2) |  |
| Right | 4,701 (50.1) | 2,681 (50.5) | 1,218 (49.9) | 751 (49.3) | 51 (41.8) |  |
| Histology |  |  |  |  |  | <0.001 |
| Ductal | 7,597 (80.9) | 4,162 (78.4) | 2,068 (84.7) | 1,268 (83.3) | 99 (81.1) |  |
| Lobular/mixed | 1,285 (13.7) | 890 (16.8) | 231 (9.5) | 152 (10.0) | 12 (9.8) |  |
| Other | 510 (5.4) | 254 (4.8) | 142 (5.8) | 103 (6.8) | 11 (9.0) |  |
| Tumour grade |  |  |  |  |  | <0.001 |
| 1 or 2 | 6,510 (69.3) | 3,948 (74.4) | 1,216 (49.8) | 1,247 (81.9) | 99 (81.1) |  |
| 3 | 2,623 (27.9) | 1,185 (22.3) | 1,164 (47.7) | 254 (16.7) | 20 (16.4) |  |
| Unknown | 259 (2.8) | 173 (3.3) | 61 (2.5) | 22 (1.4) | 3 (2.5) |  |
| Subtype |  |  |  |  |  | <0.001 |
| HR+/HER2+ | 712 (7.6) | 417 (7.9) | 222 (9.1) | 69 (4.5) | 4 (3.3) |  |
| HR+/HER2- | 6,419 (68.3) | 3,805 (71.7) | 1,344 (55.1) | 1,171 (76.9) | 99 (81.1) |  |
| HR-/HER2+ | 367 (3.9) | 241 (4.5) | 90 (3.7) | 33 (2.2) | 3 (2.5) |  |
| HR-/HER2- | 1,038 (11.1) | 399 (7.5) | 523 (21.4) | 105 (6.9) | 11 (9.0) |  |
| Unknown/DCIS | 856 (9.1) | 444 (8.4) | 262 (10.7) | 145 (9.5) | 5 (4.1) |  |
| DCIS component |  |  |  |  |  | <0.001 |
| No | 4,447 (47.3) | 2,601 (49.0) | 1,030 (42.2) | 749 (49.2) | 67 (54.9) |  |
| Yes | 4,210 (44.8) | 2,347 (44.2) | 1,172 (48.0) | 640 (42.0) | 51 (41.8) |  |
| DCIS | 717 (7.6) | 346 (6.5) | 237 (9.7) | 130 (8.5) | 4 (3.3) |  |
| Unknown | 18 (0.2) | 12 (0.2) | 2 (0.1) | 4 (0.3) | 0 (0.0) |  |
| Tumour size (mm) |  |  |  |  |  | <0.001 |
| pTis | 841 (9.0) | 424 (8.0) | 278 (11.4) | 135 (8.9) | 4 (3.3) |  |
| <30 | 7,413 (78.9) | 4,046 (76.3) | 1,945 (79.7) | 1,326 (87.1) | 96 (78.7) |  |
| 30-50 | 938 (10.0) | 691 (13.0) | 185 (7.6) | 45 (3.0) | 17 (13.9) |  |
| >50 | 45 (0.5) | 31 (0.6) | 8 (0.3) | 3 (0.2) | 3 (2.5) |  |
| pT4 | 123 (1.3) | 87 (1.6) | 23 (0.9) | 13 (0.9) | 0 (0.0) |  |
| pN/ypN |  |  |  |  |  | <0.001 |
| 0 | 6,359 (67.7) | 3,214 (60.6) | 1,718 (70.4) | 1,349 (88.6) | 78 (63.9) |  |
| 1 | 2,226 (23.7) | 1,597 (30.1) | 531 (21.8) | 75 (4.9) | 23 (18.9) |  |
| 2 | 255 (2.7) | 193 (3.6) | 54 (2.2) | 0 (0.0) | 8 (6.6) |  |
| 3 | 119 (1.3) | 79 (1.5) | 30 (1.2) | 2 (0.1) | 8 (6.6) |  |
| Unknown | 433 (4.6) | 223 (4.2) | 108 (4.4) | 97 (6.4) | 5 (4.1) |  |
| Multifocality |  |  |  |  |  | <0.001 |
| No | 7,993 (85.1) | 4,340 (81.8) | 2,105 (86.2) | 1,439 (94.5) | 109 (89.3) |  |
| Yes | 1,368 (14.6) | 948 (17.9) | 327 (13.4) | 80 (5.3) | 13 (10.7) |  |
| Unknown | 31 (0.3) | 18 (0.3) | 9 (0.4) | 4 (0.3) | 0 (0.0) |  |
| Neoadjuvant therapy |  |  |  |  |  | <0.001 |
| No | 6,589 (70.2) | 3,675 (69.3) | 1,516 (62.1) | 1,315 (86.3) | 83 (68.0) |  |
| Yes | 2,803 (29.8) | 1,631 (30.7) | 925 (37.9) | 208 (13.7) | 39 (32.0) |  |
| Surgery |  |  |  |  |  | <0.001 |
| Breast conserving therapy | 7,987 (85.0) | 4,063 (76.6) | 2,349 (96.2) | 1,485 (97.5) | 90 (73.8) |  |
| Mastectomy | 1,397 (14.9) | 1,235 (23.3) | 92 (3.8) | 38 (2.5) | 32 (26.2) |  |
| Unknown | 8 (0.1) | 8 (0.2) | 0 (0.0) | 0 (0.0) | 0 (0.0) |  |
| Direct reconstruction |  |  |  |  |  | <0.001 |
| No | 9,093 (96.8) | 5,033 (94.9) | 2,422 (99.2) | 1,520 (99.8) | 118 (96.7) |  |
| Yes | 299 (3.2) | 273 (5.1) | 19 (0.8) | 3 (0.2) | 4 (3.3) |  |
| Resection margins |  |  |  |  |  | <0.001 |
| Negative | 8,554 (91.1) | 5,186 (97.7) | 1,774 (72.7) | 1,489 (97.8) | 105 (86.1) |  |
| Focally positive | 705 (7.5) | 68 (1.3) | 605 (24.8) | 28 (1.8) | 4 (3.3) |  |
| Extensively positive | 61 (0.6) | 10 (0.2) | 39 (1.6) | 1 (0.1) | 11 (9.0) |  |
| Unknown | 72 (0.8) | 42 (0.8) | 23 (0.9) | 5 (0.3) | 2 (1.6) |  |
| Adjuvant chemotherapy |  |  |  |  |  | <0.001 |
| No | 7,798 (83.0) | 4,513 (85.1) | 1,755 (71.9) | 1,425 (93.6) | 105 (86.1) |  |
| Yes | 1,594 (17.0) | 793 (14.9) | 686 (28.1) | 98 (6.4) | 17 (13.9) |  |
| Adjuvant endocrine therapy |  |  |  |  |  | <0.001 |
| No | 4,825 (51.4) | 2,433 (45.9) | 1,247 (51.1) | 1,069 (70.2) | 76 (62.3) |  |
| Yes | 4,567 (48.6) | 2,873 (54.1) | 1,194 (48.9) | 454 (29.8) | 46 (37.7) |  |
| Adjuvant targeted therapy |  |  |  |  |  | <0.001 |
| No | 8,456 (90.0) | 4,728 (89.1) | 2,169 (88.9) | 1,442 (94.7) | 117 (95.9) |  |
| Yes | 936 (10.0) | 578 (10.9) | 272 (11.1) | 81 (5.3) | 5 (4.1) |  |
| Partial Breast Irradiation |  |  |  |  |  | <0.001 |
| No | 8,456 (90.0) | 4,988 (94.0) | 2,438 (99.9) | 968 (63.6) | 62 (50.8) |  |
| Yes | 849 (9.0) | 247 (4.7) | 0 (0.0) | 542 (35.6) | 60 (49.2) |  |
| Missing | 87 (0.9) | 71 (1.3) | 3 (0.1) | 13 (0.9) | 0 (0.0) |  |
| Type of RT institution |  |  |  |  |  | <0.001 |
| Independent | 3,055 (32.5) | 2,089 (39.4) | 712 (29.2) | 241 (15.8) | 13 (10.7) |  |
| General | 1,806 (19.2) | 942 (17.8) | 581 (23.8) | 201 (13.2) | 82 (67.2) |  |
| Academic | 4,531 (48.2) | 2,275 (42.9) | 1,148 (47.0) | 1,081 (71.0) | 27 (22.1) |  |
| Travel time by car (min) Median (IQR) | 20.0  (13.0 -27.0) | 20.0  (13.0-27.0) | 20.0  (14.0-26.0) | 19.0  (12.0-27.0) | 20.0  (14.0-26.0) | 0.38 |
| HR: Hormone receptor, IQR: interquartile range, RT: radiotherapy | | | | | | |

**Table A.4** Irradiation schedules of women in the different irradiation groups

| Schedule/group^a^ | Total | Percentage |
| --- | --- | --- |
| 15 fractions over 3 weeks |  |  |
| 15 fractions of 2.67 Gy | 5,059 | 95.3 |
| 15 fractions of 2.67 Gy, PBI | 247 | 4.7 |
| 20 fractions with boost over 4 weeks |  |  |
| 20 fractions of 2.18 Gy to the elective volumes (total 43.6 Gy), with concomitantly 20 fractions of 2.67 Gy to the tumour bed | 2,163 | 88.6 |
| 15 fractions of 2.67 Gy, with a sequential boost of five fractions of 2.67 Gy | 278 | 11.4 |
| 5 fractions |  |  |
| 5 fractions of 5.2 Gy over 1 week | 676 | 44.4 |
| 5 fractions of 5.4 Gy over 1 week | 2 | 0.1 |
| 5 fractions of 5.7 Gy over 5 weeks | 287 | 18.8 |
| 5 fractions of 6 Gy over 10 days | 16 | 1.1 |
| 5 fractions of 5.2 Gy over 1 week, PBI | 487 | 32.0 |
| 5 fractions of 5.7 Gy over 5 weeks, PBI | 23 | 1.5 |
| 5 fractions of 6 Gy PBI over 10 days, PBI | 32 | 2.1 |
| Other |  |  |
| 16 fractions of 2.67 Gy over 3 weeks | 1 | 0.8 |
| 25 fractions of 2 Gy over 5 weeks | 10 | 8.2 |
| 21 fractions of 2.03 Gy over 4 weeks, with boost | 1 | 0.8 |
| 22 fractions of 2.03 Gy over 3-5 weeks, with boost | 39 | 32.0 |
| 23 fractions of 2.03 Gy over 4.5 weeks, with boost | 4 | 3.3 |
| 10 fractions of 3.85 Gy over 2 weeks, PBI | 66 | 54.1 |
| 20 fractions of 2.18 Gy over 4 weeks, sequential boost | 1 | 0.8 |
| PBI: partial breast irradiation   1. The schedules in the irradiation groups add up to 100% | | |
